# Survival following laparoscopic versus open resection for colorectal cancer

**DOI:** 10.1007/s00384-012-1424-8

**Published:** 2012-02-09

**Authors:** Wai Lun Law, Jensen T. C. Poon, Joe K. M. Fan, Oswens S. H. Lo

**Affiliations:** Department of Surgery, The University of Hong Kong, Queen Mary Hospital, Pokfulam Road, Hong Kong, Hong Kong

**Keywords:** Outcomes of laparoscopic colorectal resection

## Abstract

**Background:**

This study aimed to compare the overall and disease specific survivals of patients who underwent laparoscopic and open resection of colorectal cancer in a high volume tertiary center.

**Methods:**

Consecutive patients who underwent elective resection for colorectal cancer (open resection, *n* = 1,197; laparoscopic resection, *n* = 814) from January 2000 to December 2009 were included. The operative details, postoperative complications, postoperative outcomes, and survival data were collected prospectively. Comparison was made between patients who had laparoscopic and open surgery.

**Results:**

The age, gender, medical morbidity, and American Society of Anesthesiologists status were similar in the two groups. Laparoscopic resection was associated with significantly less blood loss and a shorter hospital stay. The operating mortality and morbidity were significantly lower in the laparoscopic group. The qualities of the specimens in terms of the distal resection margin and the number of lymph nodes examined were not inferior in the laparoscopic group. With the median follow-up of 40.3 months, the 5-year overall survival (74.1% vs. 65.5%, *p* < 0.001) and disease specific survival (81.9% vs. 75.2%, *p* = 0.002) were significantly better in patients with non-disseminated disease in the laparoscopic group. The operative approach was an independent prognostic factor in the overall (risk ratio 1.36, 95% CI 1.093–1.700, *p* = 0.006) and disease specific (risk ratio 1.32, 95% CI 1.005–1.738, *p* = 0.048) survivals in multivariate analysis.

**Conclusion:**

Laparoscopic resection for colorectal cancer is associated with more favorable overall and disease specific survivals when compared with open resection in a high volume tertiary center.

## Introduction

Colorectal cancer is one of the most common malignancies in Western countries [1] and its incidence has also risen in Asian countries. Currently it is the second most common cancer and second leading cause of cancer death in Hong Kong [2]. Surgical resection has remained the mainstay treatment for colorectal cancer. However, the operation is a major undertaking and is associated with significant morbidity, especially is elderly patients with concomitant medical conditions. Laparoscopic resection has been reported to improve the short-term outcomes in terms of less postoperative pain and analgesic requirement, quicker recovery and a shorter hospital stay [3–6]. Data from randomized trials comparing open and laparoscopic colon resection also demonstrated that survival after laparoscopic resection was not inferior to open resection [7–10]. Whether the advantage of fewer complications and better short-term outcomes can be translated to a better survival in patients with cancer is controversial. More favorable survival in patients who underwent laparoscopic resection has been demonstrated in a randomized trial [10] and a population study [11]. We previously also reported better overall survival in patients who underwent laparoscopic colon and rectal resection in series of smaller number of patients [12, 13]. In this study, we would like to confirm the findings with a cohort of larger number of patients with longer follow-up. The current study aimed to evaluate the outcomes including survival of consecutive patients who underwent laparoscopic resection for colorectal malignancy in a high volume tertiary center. Comparison of the outcomes with those patients who underwent open resection performed during the same period of time was performed.

## Methods

Laparoscopic resection has become widely applied in the authors’ department since 2000. During the study period from 2000 to 2009, the choice of surgical approach was decided mainly by the surgeons’ experience and patients’ preference with all the risk of both approaches discussed with the patients. Since 2008, when the senior author was in charge of the Division of Colorectal Surgery, laparoscopic resection was offered to all suitable patients who planned to have elective surgery for colorectal cancer unless contraindicated.

The operation techniques of laparoscopic surgery were described in the previous publications. A standardized medial to lateral approach was adopted from 2004 [14]. The majority of patients underwent laparoscopic assisted resection with three to five ports and the retrieval of the specimen was performed through an abdominal incision. In those patients who underwent laparoscopic abdominoperineal resection or low anterior resection with coloanal anastomosis, the specimen would be retrieved through the perineum or anus. Transvaginal retrieval of the specimen was performed in selected female patients who underwent commitment hysterectomy. From 2008, some selected patients were operated on with robotic-assisted resection or single incision laparoscopic surgery [15].

During the study period, the patients who underwent laparoscopic or open resection had similar preoperative workup and preparation for surgery. The postoperative management and the policies of adjuvant therapy were similar in all the patients, regardless of the surgical approach.

All the operations were performed or supervised by specialists in the Division of Colorectal Surgery. In the early period, two staff surgeons performed or supervised the majority of laparoscopic operations and they also performed the open operations. From 2004, with the departure of one laparoscopic surgeon, the senior surgeon supervised and performed the majority of the laparoscopic resection until the other staff members were trained to perform laparoscopic colectomy in a standardized technique. All the laparoscopic rectal resections are still performed or under the supervision by the senior author.

### Definition

Operative mortality was defined as deaths that occurred within 30 days following the primary operation. Operative morbidities were defined as complications that contributed to prolonged hospital stay or led to additional interventions or procedures.

Conversion was defined as the need for prematurely making the abdominal incision for bowel mobilization and/or vascular control. The necessity for an abdominal incision to deal with any intra-operative complication was also considered conversion.

### Data collection and statistical analysis

Data on the patients’ demographics, medical comorbidities, locations of the tumors, operative details, postoperative outcomes, and follow-up status were collected prospectively and entered into a database for colorectal malignancy. In the comparison of data on patients with laparoscopic and open resection, the analysis was performed according to the intention to treat principle. Patients with conversion were analyzed in the laparoscopic resection group.

Comparison of the categorical or ordinal variables was performed using chi-square test or Fisher’s exact test where appropriate. Continuous variables were presented in median values and interquartile ranges. Comparison was performed using Mann–Whitney *U* test. Survival analysis was performed after excluding patients who died within 30 days after the surgery and who had stage IV disease. Survival was analyzed using Kaplan–Meier method and comparison of variables was performed with log rank test. Multivariate analysis was performed with Cox regression using variables found to be statistically significant in univariate analysis. *p* values of less than 0.05 were regarded statistically significant.

## Results

After excluding those patients who underwent emergency surgery, operations without resection and local excision of rectal cancer, 2,011 patients were included in the current study. They underwent laparoscopic or open radical resection for adenocarcinoma of colon or rectum. There were 1,157 men (57.5%) and the median age was 71 years (range, 22–96 years). In 911 patients (45.3%), the tumors were located at the rectum or rectosigmoid. All operations were performed on an elective setting and patients with emergency operations were excluded. Thirty-nine patients, who had obstructing left-sided colorectal cancer, were included. Twenty-seven had metallic stent insertion prior to resection and 12 had prior colostomy for rectal cancer so that resection can be performed on an elective setting, usually after neoadjuvant therapy.

The operative mortality and morbidity rates of all the patients were 1.3% and 23.7%, respectively. Laparoscopic resection was performed in 814 patients (40.5%). In those patients with laparoscopic resection, 58 required conversion and the conversion rate was 7.1%. The 30-day mortality was 0.5% and the complication rate was 17.3% in those patients who had laparoscopic resection. Conversion was associated with significantly more blood loss, higher complication rate, and a longer hospital stay when compared with successful laparoscopic procedures.

Comparison between laparoscopic and open operation is shown in Table 1. Laparoscopic resection was associated with significantly less blood loss, a lower incidence of postoperative complications and mortality as well a shorter hospital stay. The cardiac, pulmonary complications as well as postoperative ileus were also significantly fewer in patients who underwent laparoscopic resection (Table 2).

**Table 1:** Comparison of patient with open and laparoscopic resection for colorectal cancer

	Open	Laparoscopic	*p* values
	*n* = 1197	*n* = 814	
Male/female	702:495	455:359	0.232
Median age (years)	71 (61–77)^a^	70 (61–78)^a^	0.472
Colon	631 (52.7%)	468 (57.5%)	0.036
Rectum	566 (47.3%)	346 (42.5%)	
Presence of medical diseases	687 (57.4%)	473 (58.1%)	0.748
ASA class 3–5	261(21.8%)	153 (18.8%)	0.081
Operating time (min)	130 (105–169)^a^	180 (141–218)^a^	<0.001
Blood loss (ml)	200 (100–450)^a^	100 (50–200)^a^	<0.001
Complications	335 (28.0%)	141 (17.3%)	<0.001
Operative mortality (30 days)	23 (1.9%)	4 (0.5%)	0.005
Stage I	148 (12.4%)	152 (18.6%)	0.001
Stage II	476 (39.8%)	285 (35.0%)	
Stage III	400 (33.4%)	246 (30.2%)	
Stage IV	173 (14.5%)	131 (16.1%)	
Median distal margin (cm)	4.0 (2.5–6.0)^a^	4.0 (2.5–6.0)^a^	0.374
Size of tumors (cm)	4.0 (3.0–6.0)^a^	4.0 (3.0–5.0)^a^	0.005
Differentiation			<0.001
Well	65	48	
Moderate	1,009	725	
Poor	123	41	
Lymphovascular permeation	382	239	0.238
Median no. of lymph nodes examined	11 (7–16)	13 (8–18)^a^	*p* < 0.001
Perineural invasion	168	100	0.285
Median hospital stay	8 (6–11)^a^	5 (4–8)^a^	<0.001
Reoperation	41 (3.4%)	26 (3.2%)	0.802

*ASA* American Society of Anesthesiology class

^a^Figures in parenthesis are interquartile range

**Table 2:** Comparison of postoperative complications between open and laparoscopic resection

	Open	Laparoscopic	*p* values
	*n* = 1197	*n* = 814	
Cardiac complications	59 (4.9%)	21 (2.6%)	0.008
Pulmonary complications	64 (5.3%)	22 (2.7%)	0.005
Deep vein thrombosis/pulmonary embolism	9 (0.8%)	5 (0.6%)	0.791
Ileus	82 (6.9%)	32 (3.9%)	0.006
Wound infection	52 (4.3%)	25 (3.1%)	0.156
Anastomotic leak	29 (2.4%)	15 (1.8%)	0.439
Urological complications	52 (4.3%)	36 (4.4%)	1.000

Regarding the resected specimens, there is no difference in the distal margin. Patients with open operations tended to have bigger tumors although the median size of tumors was 4 cm in both groups. There were significantly more lymph nodes examined in the laparoscopic group (Table 1).

The median follow-up period of the patients was 40.3 months. In those patients with stage IV disease, there was no difference in survival between those with laparoscopic or open resection.

**Fig. 1:**
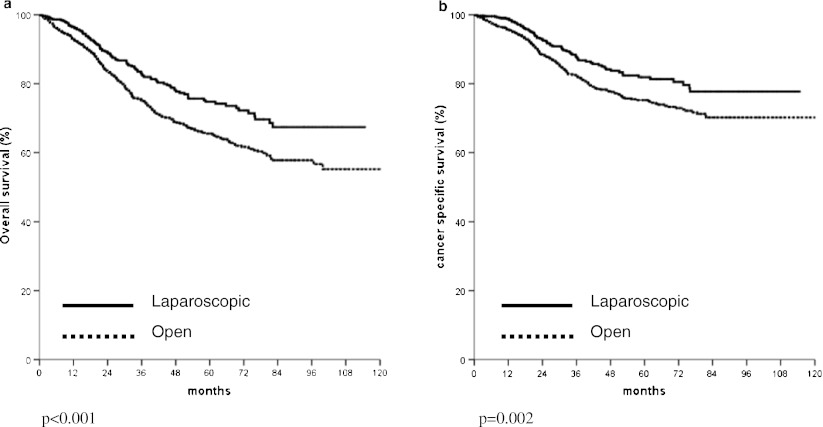
**a** Comparison of overall survivals of patients who underwent laparoscopic and open colorectal resection. **b** Comparison of cancer specific survivals of patients who underwent laparoscopic and open colorectal resection

**Fig. 2:**
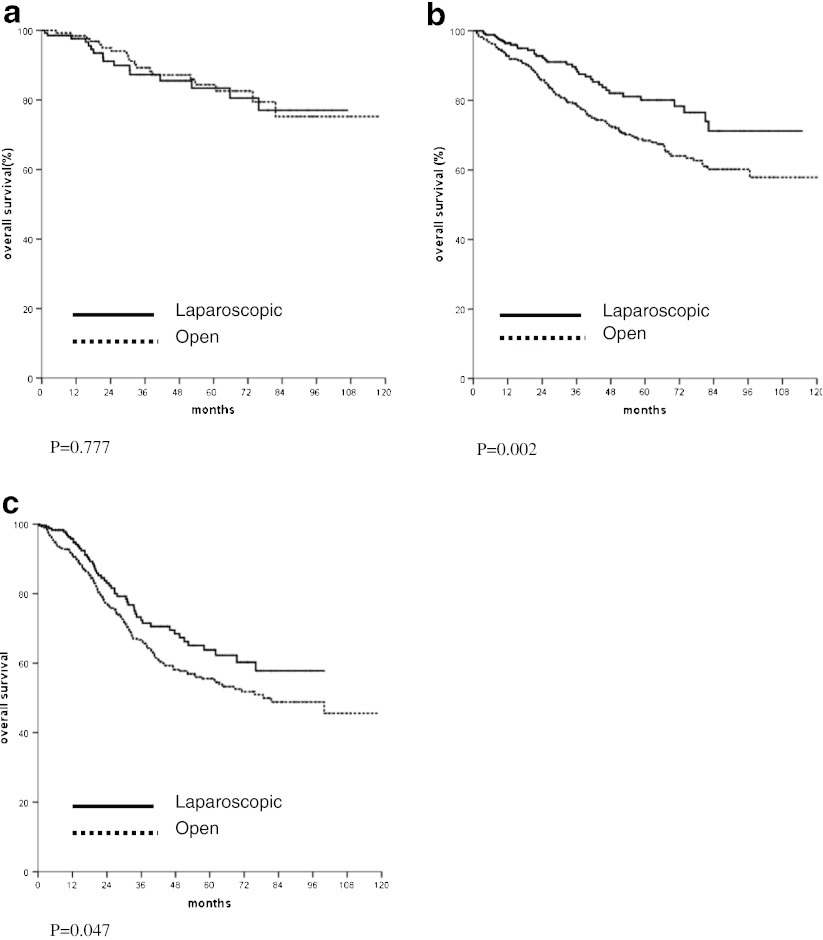
**a** Comparison of overall survival of patients with stage I cancer. **b** Comparison of overall survival of patients with stage II cancer. **c** Comparison of overall survival of patients with stage III cancer

**Fig. 3:**
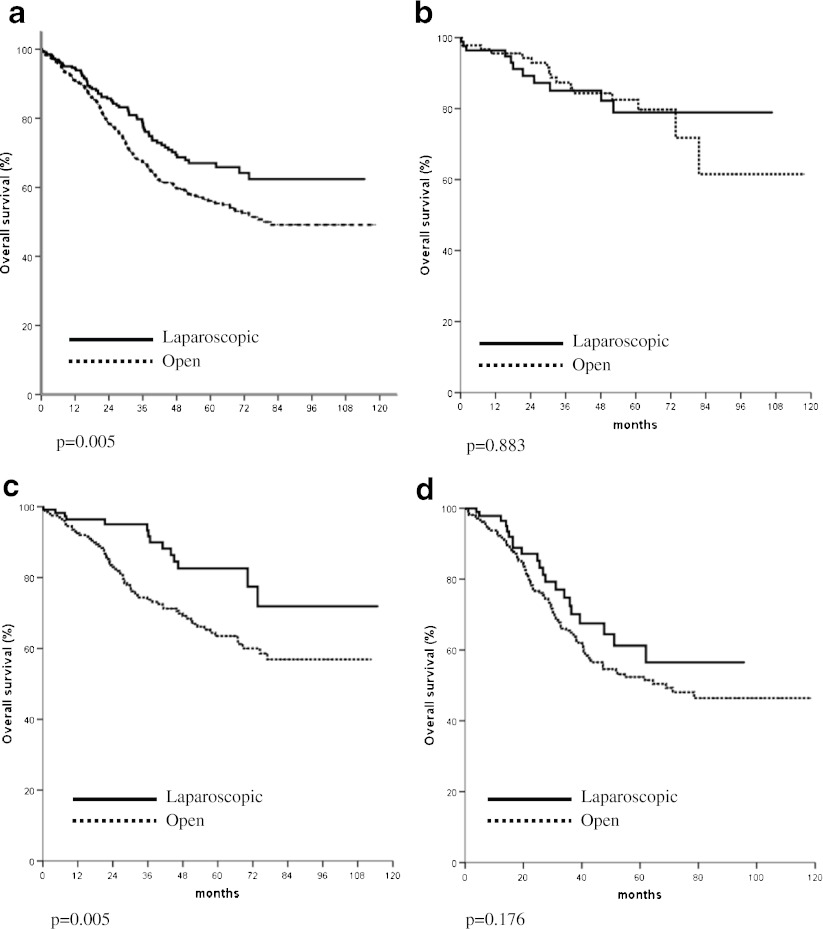
**a** Comparison of overall survival of patients with rectal cancer (all stages). **b** Comparison of overall survival of patients with stage I rectal cancer. **c** Comparison of overall survival of patients with stage II rectal cancer. **d** Comparison of overall survival of patients with stage III rectal cancer

**Fig. 4:**
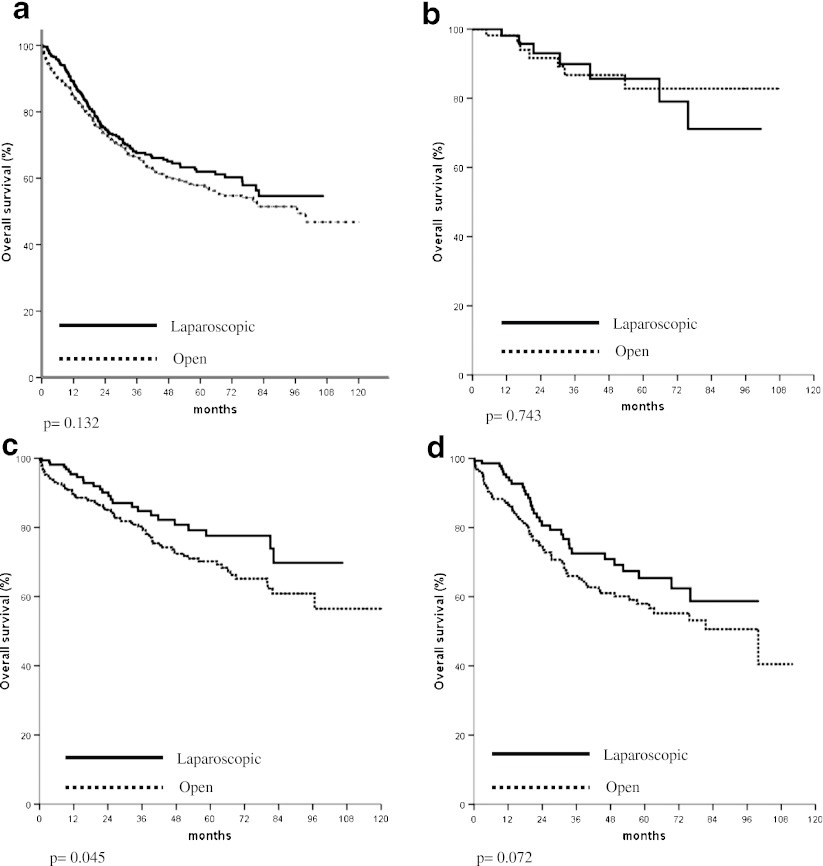
**a** Comparison of overall survival of patients with colon cancer (all stages). **b** Comparison of overall survival of patients with stage I colon cancer. **c** Comparison of overall survival of patients with stage II colon cancer. **d** Comparison of overall survival of patients with stage III colon cancer

In the analysis of survival of patients with non-disseminated disease, the overall and cancer specific survivals of those treated with laparoscopic resection were significantly better than those treated with open operation (Fig. 1a, b). The comparison of overall survivals of patients with stage I, II, and III diseases is shown in Fig. 2a–c, respectively. In patients with stage II or stage III cancer, significantly better overall survival was found in those who underwent laparoscopic resection. The comparison of overall survivals of patients with rectal and colon cancer are shown in Figs. 3a and 4a, respectively. While there was no difference in survival in patients with colon cancer, laparoscopic resection was associated with better survivals in patients with rectal cancer. The overall survivals of patients with different stages of diseases of rectal cancer and colon cancer are shown in Figs. 3b–d and 4b–d, respectively. There was no difference in survival in patients with stage I disease. The improvement in survival in the laparoscopic group occurred mainly in patients with stage II disease (both colon and rectal cancer). There was also a trend towards better survival in patients with stage III disease, although it did not show any statistical significance.

Other factors that influence overall and disease specific survivals in univariate analysis are shown in Table 3. Besides, the surgical approach, the survival is most related to the histological findings of the tumor. The results of multivariate analysis of overall and disease specific survivals are shown in Tables 4 and 5, respectively. On multivariate analysis, laparoscopic resection remained one of the independent factors associated with better overall survival as well as disease specific survival.

**Table 3:** Univariate analysis of overall survival and cancer specific survival in patients with colorectal resection

	Overall survival	*p* values	Cancer specific survival	*p* values
Male	66.3%	0.042	76.2	0.222
Female	71.6%		79.1	
Colon	70.9%	0.344	81.5	0.005
Rectum	66.4%		73.7	
Laparoscopic	74.7%	<0.001	81.9	0.002
Open	65.5%		75.2	
Stage I	83.0%	<0.001	93.6%	<0.001
Stage II	72.1%		81.7%	
Stage III	58.1%		66.8%	
Age >70	63.3%	<0.001	77.5	0.398
Age ≤ 70	73.9%		77.8	
Presence of medical diseases		0.042		0.324
No	71.9%		76.5	
Yes	66.2%		78.3	
Perineural invasion		<0.001		<0.001
No	71.0%		80.6	
Yes	44.0%		46.5	
Lymphovascular permeation		<0.001		<0.001
No	72.7%		82.3	
Yes	55.8%		62.6	
Differentiation		0.003		<0.001
Well	76.2%		90.0	
Moderate	68.7%		77.7	
Poor	61.2%		64.9	
No complications	71.6%	<0.001	79.8	<0.001
Complications	59.1%		70.1

**Table 4:** Multivariate analysis of factors affecting overall survival

	Risk ratio	*p* value	95% confidence interval
Open operation	1.36	0.006	1.093–1.700
Male	1.20	0.077	0.980–1.461
Age >70	1.72	<0.001	1.399–2.110
Presence of medical diseases	1.10	0.357	0.897–1.351
Stage of disease	1.56	<0.001	1.335–1.832
Differentiation	1.26	0.095	0.961–1.651
Perineural invasion	1.55	0.003	1.161–2.075
Lymphovascular invasion	1.23	0.079	0.976–1.548
Postoperative complication	1.60	<0.001	1.299–1.969

**Table 5:** Multivariate analysis of factors affecting cancer specific survival

	Risk ratio	*p* value	95% confidence interval
Open operation	1.32	0.048	1.005–1.738
Stage of disease	1.83	<0.001	1.489–2.246
Differentiation	1.66	0.002	1.207–2.288
Perineural invasion	1.89	<0.001	1.372–2.611
Lymphovascular invasion	1.37	0.024	1.044–1.811
Postoperative complication	1.57	0.001	1.215–2.041
Rectal cancer	1.28	0.048	1.003–1.635

## Discussion

Colorectal cancer is a common malignancy, which usually occurs in the elderly age group. Many of the patients have significant medical co-morbidities, which affect the operative outcomes. Laparoscopic resection revolutionized the treatment of colorectal malignancy in recent years. With the introduction of laparoscopic resection, favorable operative outcomes in terms of less pain, less analgesic requirement, quick recovery of the gastrointestinal tract, and a shorter hospital stay were demonstrated in most randomized controlled trials [3–6].

The current study showed that laparoscopic surgery not only improved the short-term outcomes of patients with colorectal cancer, but it also led to lower postoperative morbidity and mortality as well as improvement in both overall and cancer specific survivals in a center with a high volume of cases. Despite improvement in many parameters, which assessed postoperative outcomes, most individual trials were unable to demonstrate a reduction in complication rate or mortality. Nevertheless, a significant reduction in mortality rate and a trend towards a lower morbidity could be demonstrated in a meta-analysis [16]. Moreover, a lower complication rate was shown in population studies which included a large number of patients [17]. The inability to demonstrate improvement in most randomized trials might be due to the fact that the trials were not powered to show the difference. In clinical practice, both in the community as well as individual center with a large number of patients, laparoscopic surgery helped to reduce the complication rate. Another reason for not able to demonstrate a lower complication rate following laparoscopic surgery is likely due to the high conversion rates most of the published trials. We believe that a low conversion rate is important to generate the benefit of laparoscopic surgery as most of the studies are analyzed with the intention to treat principle. There are controversies on whether conversion is associated with poor outcome. In the CLASICC trial, the conversion was associated with worse outcome when compared with open operation [6]. Other studies showed that the outcome was not worse with open operation [18, 19]. However, in cases of conversion, the outcome would at best be similar to open resection and the benefit of operation regarding the cardiopulmonary complications, ileus and wound complication of laparoscopic surgery cannot be derived. Lacy et al. demonstrated fewer complications and better survival in patients with laparoscopic resection and the presence of a low conversion rate is important to obtain the beneficial results [3].

The survival is the most important outcome to assess treatment success for malignant disease. In colorectal cancer, both the overall survival and cancer specific survival are important as many patients are elderly and death might be due to other medical diseases, which might be related to the operation. We demonstrated both a significantly better overall and cancer specific survivals in patients who underwent laparoscopic resection. The approach of surgery was shown to be an independent significant factor of both overall and cancer specific survival. This confirmed our previous findings on the improved survival in patients with laparoscopic colon and rectal resection with a larger patient population. The improvement occurred mainly both stage II and stage III cancer and occurred both in colon and rectal cancer. This is different from multicenter randomized studies [7–9], which showed equivalent survival in patients with open and laparoscopic resection. We postulated that most of these randomized trials such as the COST trial [4] were initially planned as non-inferiority studies and were not powered to show the difference in survival.

We are not the only group, which reported superior survival in laparoscopic resection. Lacy et al. reported better survival in laparoscopic resection in a single center randomized trial and the better survival was mainly in the group of patients with stage III cancer [10]. Capussotti et al. also found that in patients with stage III colon cancer laparoscopic resection was associated with a significantly disease free and cancer related survival [20]. We also demonstrated better overall survival in stage II and stage III cancer. In the study by Bilimora et al. using the National Cancer data, better survival was found in the patients with laparoscopic surgery [11]. In case of rectal cancer, Laurant et al. also demonstrated better survival in patients with laparoscopic resection, but there was no difference in cancer free survival [21]. Thus improvement of survival can be achieved with the laparoscopic approach.

One of the reasons accounting for the better survival might be the better immunological response in patients who underwent laparoscopic surgery. This has been demonstrated in many studies on the inflammatory markers after laparoscopic surgery [22, 23]. The association of cytokines such as interleukin 6 and VEGF, which was produced significantly more after open surgery, with tumor recurrence has been demonstrated in animal models. The less release of cytokines such as interleukin 6 and VEGF in the postoperative period has been demonstrated in laparoscopic surgery [24]. This might also contribute to the better oncologic outcome in laparoscopic resection. Moreover, the lower complication rate associated with laparoscopic resection might also contribute to the better survival. Khuri and colleagues showed with the NSQIP data that the presence of postoperative complications adversely affected the long-term survival of patients in eight operations, which included colorectal resection [25]. The presence of postoperative complications has also been demonstrated to affect the survival of patients who underwent resection for colorectal, esophageal and liver cancer [26–29]. Thus the lower complication rate associated with laparoscopic resection might be the reason accounting for the better survival. This is exemplified by Lacy et al.’s trial in which a significant lower complication rate as well as better survival were demonstrated in the patients with laparoscopic resection.

Admittedly, the study is not a randomized trial and biases in the selection of patients for laparoscopic procedures were unavoidable. It could be shown that there were more patients with earlier cancer in the laparoscopic group. The size of the tumor was also larger in the open group. However, other parameters including the distal resection margin as well as the number of lymph nodes examined were not inferior in laparoscopic resection. The surgical approach remained an independent significant factor associated with for better survival. Moreover, the difference in survival between the laparoscopic and open resection could be demonstrated in analysis according to the stage of the disease. This study also highlighted the impact of laparoscopic resection on the outcomes of treatment of colorectal cancer in a high volume center. With the wider application of laparoscopic resection for colorectal malignancy worldwide, it is expected that more data from large volume centers or from population studies can give more information on the impact of survival with the shift to laparoscopic surgery.

## Conclusion

In the current study, laparoscopic resection for colorectal cancer was shown not only to be associated with better short-term results, but the overall and disease specific survivals were also better when compared to open resection.
